# Trait self-esteem and neural activities related to self-evaluation and social feedback

**DOI:** 10.1038/srep20274

**Published:** 2016-02-04

**Authors:** Juan Yang, Xiaofan Xu, Yu Chen, Zhenhao Shi, Shihui Han

**Affiliations:** 1 Faculty of Psychology, Southwest University, Chongqing, 400715, China; 2 The Annenberg Public Policy Center, University of Pennsylvania, Philadelphia, PA 19104-3806, USA; 3 Department of Psychology, PKU-IDG/McGovern Institute for Brain Research, Beijing Key Laboratory of Behavior and Mental Health, Peking University, Beijing, 100871, China

## Abstract

Self-esteem has been associated with neural responses to self-reflection and attitude toward social feedback but in different brain regions. The distinct associations might arise from different tasks or task-related attitudes in the previous studies. The current study aimed to clarify these by investigating the association between self-esteem and neural responses to evaluation of one’s own personality traits and of others’ opinion about one’s own personality traits. We scanned 25 college students using functional MRI during evaluation of oneself or evaluation of social feedback. Trait self-esteem was measured using the Rosenberg self-esteem scale after scanning. Whole-brain regression analyses revealed that trait self-esteem was associated with the bilateral orbitofrontal activity during evaluation of one’s own positive traits but with activities in the medial prefrontal cortex, posterior cingulate, and occipital cortices during evaluation of positive social feedback. Our findings suggest that trait self-esteem modulates the degree of both affective processes in the orbitofrontal cortex during self-reflection and cognitive processes in the medial prefrontal cortex during evaluation of social feedback.

People strive to feel good about themselves, or seek to maintain their self-esteem, and this is a fundamental human nature^1^,^2^. From the *intrapersonal* perspective, self-esteem has been viewed as evaluation of one’s own goodness or worth^3^ or a personal assessment of how well one is doing in areas that the individual regards as important^1^. An *interpersonal* perspective, however, suggests that people’s thoughts and feelings about themselves reflect, in part, how they believe they are perceived and evaluated by others^4^. The sociometer theory proposes that social feedback from others produces a strong effect on self-esteem because the self-esteem system itself is a subjective monitor or gauge of the degree to which the individual is being accepted by other people^2^,^5^.

It has long been suggested that self-esteem reflects the need for both self-respect and respect from others^6^. Behavioral evidence has revealed that people with high self-esteem, who believe that they are socially approved, rate themselves more positively, whereas those with low self-esteem, who doubt their social worth, rate themselves lower on socially valued traits^3^. However, to date, it remains unknown whether and how neural activities related to one’s own and others’ opinions about the self are associated with dispositional self-esteem. On the one hand, Yang *et al.* (2012) found that levels of trait self-esteem, estimated by the Rosenberg self-esteem scale^7^, were negatively associated with the neural activity in the dorsal anterior cingulate cortex (ACC) in response to self-evaluation compared to other-evaluation^8^. On the other hand, Eisenberger and colleagues found that neural responses in the dorsal ACC, bilateral anterior insula and dmPFC to the attitude toward social feedback about the self were negatively associated with state self-esteem (which was estimated by measuring emotional states in response to each feedback)^9^.

Taken together, these observations suggest that the neural activities in different brain regions related to one’s own and others’ opinions about the self are separately associated with their self-esteem. However, the previous studies employed different tasks and scanned different cultural populations, it is unclear whether the distinct associations between self-esteem and brain activity arose from the different tasks or subject samples. Neural activity in response to personality traits that are determined a priori to be positive or negative mainly reflected participants’ neural response related to the task^10^,^11^, while neural activity related to participants’ individualized response to personality traits mainly reflected participants’ neural response associated with their attitude^9^,^12^. The effect of different cultural samples is also possible given the substantial evidence of cultural influences on brain activity involved in multiple cognitive and affective processes^13^,^14^. To clarify these, the present study recruited the same cultural sample (i.e., Chinese) and employed the same evaluation task. We tested whether and how one’s self-esteem is associated with the neural activity during evaluations of one’s own personality trait and evaluation of others’ opinion about one’s own personality its. During fMRI scanning, participants were asked to reflect on the self or a celebrity and to reflect on social feedback to the self or to a celebrity by responding on a 4-point scale. Neural activity underlying the reflection tasks was estimated by contrasting reflection on the self versus other or by contrasting reflection on social feedback about the self or a celebrity. Neural activity related to attitude was defined by regressing brain activity to participants’ responses during self-reflection and during judgments on social feedback. This design allowed us to examine whether trait self-esteem can be associated with neural activity related to both self-evaluation and social feedback. Moreover, as trait self-esteem was defined as the tendency to evaluate oneself positively rather than negatively^15^, we were also interested in the associations between self-esteem and the neural activity related to both evaluation of positive traits of the self and attitudes toward the positive traits of the self.

Self-esteem is considered to be a relatively enduring characteristic that possesses both affective and cognitive components^16^. The way people represent themselves in comparison with others, and the role of affective processes in such representations are matters of significant interest to a social cognitive and affective neuroscience of trait self-esteem^11^. On the one hand, research on self-esteem that focused on associated intrapersonal experiences (self-evaluation) defined self-esteem as one’s feeling about the self^17^. Self-esteem, at its roots, is an affective, top-down, internal experience. That is, people feel either good or bad about the self (affect), which then guides evaluation (cognition), and drives action (behavior)^16^. On the other hand, there is also a long history of accounting for self-esteem’s interpersonal influences^18^. From this perspective, people processed others’ reactions of inclusion and exclusion in an automatic cognitive mechanism^5^. In other words, how an individual believes that he or she is viewed in the eyes of others is critical in forming of self-esteem. As outlined above, we assumed that neural activity in brain regions involved in affective process would be associated with self-esteem during the self-evaluation process, whereas neural activity in brain regions involved in cognitive process would be associated with self-esteem during the social feedback process.

## Materials and Methods

### Participants

Twenty-nine healthy college students (15 males) participated in this study as paid volunteers (mean age ± SD = 20.1 ± 1.4). All reported no history of psychiatric or neurological disorders, significant physical illness, head injury, or alcohol/drug use, were recruited from Southwest University (Chongqing, China). Four participants were excluded from data analysis due to excessive head motion during scanning and 25 participants (13 males) were included in fMRI data analysis [mean age ± SD = 20.1 ± 1.3]. All participants gave informed consent prior to scanning. The experiments were carried out in accordance with relevant guidelines and regulations. The protocol for this study was approved by the Ethics Committee of Southwest University.

### Questionnaire

Before scanning, participants completed the Rosenberg self-esteem scale (RSE)^7^ to assess their overall evaluation of self-worth. The RSE consists of 10 items such as ‘I feel I do not have much to be proud of’ or ‘On the whole, I am satisfied with myself’, which are coded on a 4-point scale ranging from 1 (strongly disagree) to 4 (strongly agree). Negative items were reversed scored.

### Stimulus and Procedure

To create a context of social feedback for scanning, we first asked 120 students to evaluate five of their classmates as well as a celebrity (i.e., Xiang Liu, a well-known Chinese athlete) on 10 personality-trait adjectives (1 = strongly disagree; 4 = strongly agree). Twenty-nine of these students were then randomly recruited for fMRI scanning. 336 personality-trait adjectives (half positive and half negative) were selected from an established personality trait adjective pool^19^ for fMRI scanning. Each adjective consists of two Chinese characters. Stimuli were presented through a projector onto a rear-projection screen located at the subject’s head.

**Figure 1:**
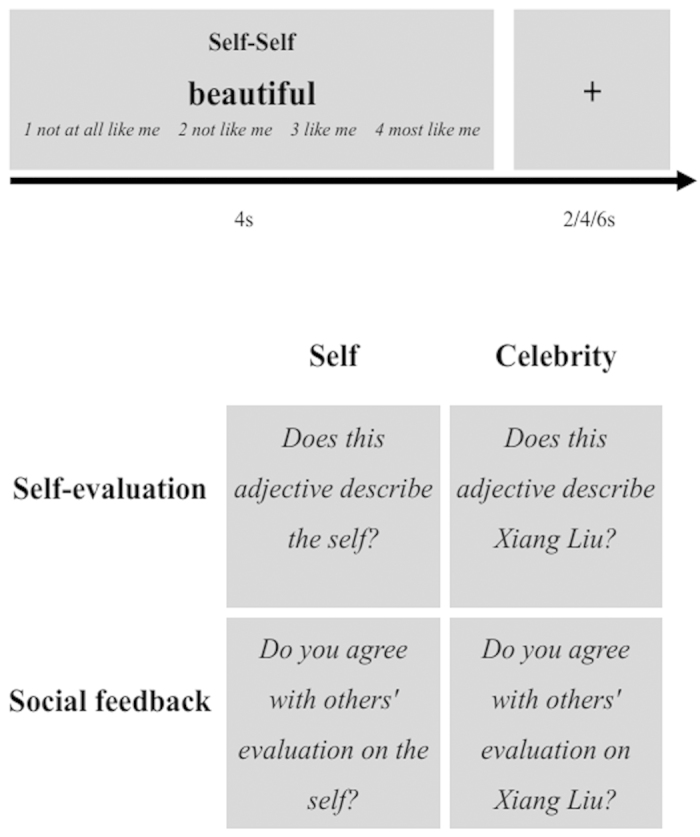
Experimental procedure (top) and experimental conditions (bottom) used in the fMRI study. The conditions varied according to the Target of the evaluation (self versus Celebrity) and to the Task of the evaluation (self-evaluation versus social feedback).

A mixed blocked/event-related design was used in 7 functional scans. Each scan consisted of 8 blocks of trials and 2 successive blocks were intervened by a 10-s grey screen. Each block consisted of 6 trials. On each trial a black trait adjective was presented for 4 s at the center of a grey screen, which was followed by a fixation with a duration of 2, 4, or 6 s. Experimental procedure and experimental conditions used in the fMRI study are listed in Fig. 1. There were 4 different tasks and participants performed one task in each block of trials. The self-evaluation task asked participants to evaluate “Does this adjective describe the self?” by pressing on of 4 buttons (1 = not at all like me, 4 = most like me). The celebrity-evaluation task asked participants to indicate “Does this adjective describe Xiang Liu?” During the task of evaluating feedback on the self, participants were told that they were presented with a number of trait adjectives that were used by their classmates to describe them. They had to indicate “Do you agree with others’ evaluation on the self?” by a button press (1 = strongly disagree, 4 = strongly agree) on each trait adjective. During the task of evaluating others’ feedback on the celebrity, participants were told that the celebrity had been described by their classmates using a number of trait adjective and they had to indicate “Do you agree with others’ evaluation on Xiang Liu?” by a button press (1 = strongly disagree, 4 = strongly agree) on each trait adjective. There were 42 positive and 42 negative adjectives for each task. Stimuli were classified into 8 conditions, i.e., evaluation of positive traits of the self (EPS), evaluation of negative traits of the self (ENS), evaluation of positive traits of the celebrity (EPC), evaluation of negative traits of the celebrity (ENC), evaluation of others’ positive feedback on the self (EPFS), evaluation of others’ negative feedback on the self (ENFS), evaluation of others’ positive feedback on the celebrity (EPFC), evaluation of others’ negative feedback on the celebrity (ENFC).

### fMRI Data Acquisition

Images were acquired in a 3T Siemens TRIO MRI scanner. Functional data comprised 1680 volumes acquired with T2*-weighted gradient echo planar imaging (EPI) sequences. We obtained 32 echo planar images per volume sensitive to blood oxygenation level-dependent (BOLD) contrast (TR = 2000 msec; TE = 30 msec; 3 mm × 3 mm in-plane resolution; Field of View [FOV] = 192 mm × 192 mm). Slices were acquired in an interleaved order and oriented parallel to the AC-PC plane, with thickness of 3 mm, 0.99 mm gap. High-resolution T1-weighted 3D fast –field echo (FFE) sequences were obtained for anatomical reference (176 slices, TR = 1900 msec; TE = 2.52 msec; slice thickness = 1 mm; FOV = 250 mm × 250 mm; voxel size = 1 mm × 1 mm × 1 mm).

### fMRI Data Analysis

Data were analyzed using Brain Voyager QX v2.3 (Brain Innovation, The Netherlands). Functional scans were realigned within and across runs to correct for head motion, and co-registered with each participant’s anatomical data. Functional data were then normalized into standard stereotactic Talairach space, resliced into a voxel size of 3 × 3 × 3 mm^3^ and smoothed with an 8 mm Gaussian kernel to increase signal-to-noise ratio. Event-related effects were estimated using the general linear model and employing a canonical hemodynamic response function convolved with the experimental design. Fixed effect analyses were first performed to estimate effect at each voxel and to compare regionally specific effects in individual participants using linear contrast. Group analyses were then conducted using random-effects models to enable population inferences. Inference of statistical significance used uncorrected *p* value *p* < 0.005, in regions encompassing at least 20 voxels^20^.

#### Modeling of self-related contrasts during the self-evaluation task

Brain activations associated with evaluation of one’s own traits was estimated by contrasting (EPS + ENS) versus (EPC + ENC). The contrast of (EPS−EPC) versus (ENS–ENC) was calculated to define brain regions involved in evaluation of positive traits of the self. Moreover, to identify whether participants’ trait self–esteem can modulate their brain activations related to evaluation of one’s own traits, self–esteem scores derived from the RSE questionnaire were entered as a regressor in a whole–brain regression analysis to assess its associations with the contrast value of (EPS + ENS) versus (EPC + ENC) or (EPS–EPC) versus (ENS–ENC), respectively.

Further, brain activations related to participants’ attitude about the self were estimated by regressing participants’ rating of each trait adjective on a 4–point scale (1 = strongly disagree, 4 = strongly agree). Brain activations that showed linear relationships with increasing rating in evaluating traits of the self were calculated. The contrasts of (EPS +ENS) versus (EPC + ENC) or (EPS–EPC) versus (ENS–ENC) were conducted to assess brain activations related to attitudes toward the self or attitudes toward the positive traits of the self, respectively. Moreover, to identify whether people’s trait self–esteem could modulate the brain regions that showed a linear relationship with increasing rating in evaluating traits of the self or positive traits of the self, the self–esteem scores derived from the RSE questionnaire were entered as a regressor in a whole–brain regression analysis to assess its associations with the contrast value of (EPS + ENS) versus (EPC + ENC) or (EPS–EPC) versus (ENS–ENC), respectively.

#### Modeling of self–related contrasts during the social feedback task

Brain activations related to evaluation of others’ feedback on the self was estimated by contrasting (EPFS + ENFS) versus (EPFC + ENFC). The contrast of (EPFS–EPFC) versus (ENFS–ENFC) was calculated to define brain regions engaged in evaluation of others’ positive feedback on the self. Moreover, to identify whether participants’ trait self–esteem can modulate their brain activations involved in evaluation of social feedback on the self or positive social feedback on the self, a whole–brain regression analysis of the contrast value of (EPFS + ENFS) versus (EPFC + ENFC) or the contrast value of (EPFS–EPFC) versus (ENFS–ENFC) were conducted with self–esteem score as a regressor.

Further, brain activations related to participants’ attitude about social feedback were estimated by regressing participants’ rating of each trait adjective on a 4–point scale (1 = strongly disagree, 4 = strongly agree). The contrast of (EPFS + ENFS) versus (EPFC + ENFC) was then conducted to assess brain activations related to attitudes toward the social feedback on the self. In addition the contrast of (EPFS–EPFC) versus (ENFS–ENFC) was used in the regression analyses to examine brain activations related to attitude toward positive social feedback on the self. Moreover, to identify whether people’s trait self–esteem could modulate their brain regions that showed a linear relationship with increasing rating in evaluating other’s feedback on the self or other’s positive feedback on the self, we regressed self–esteem score with the contrast value of (EPFS + ENFS) versus (EPFC + ENFC) or the contrast value of (EPFS–EPFC) versus (ENFS–ENFC), respectively.

## Results

### Behavioral Performances

**Figure 2:**
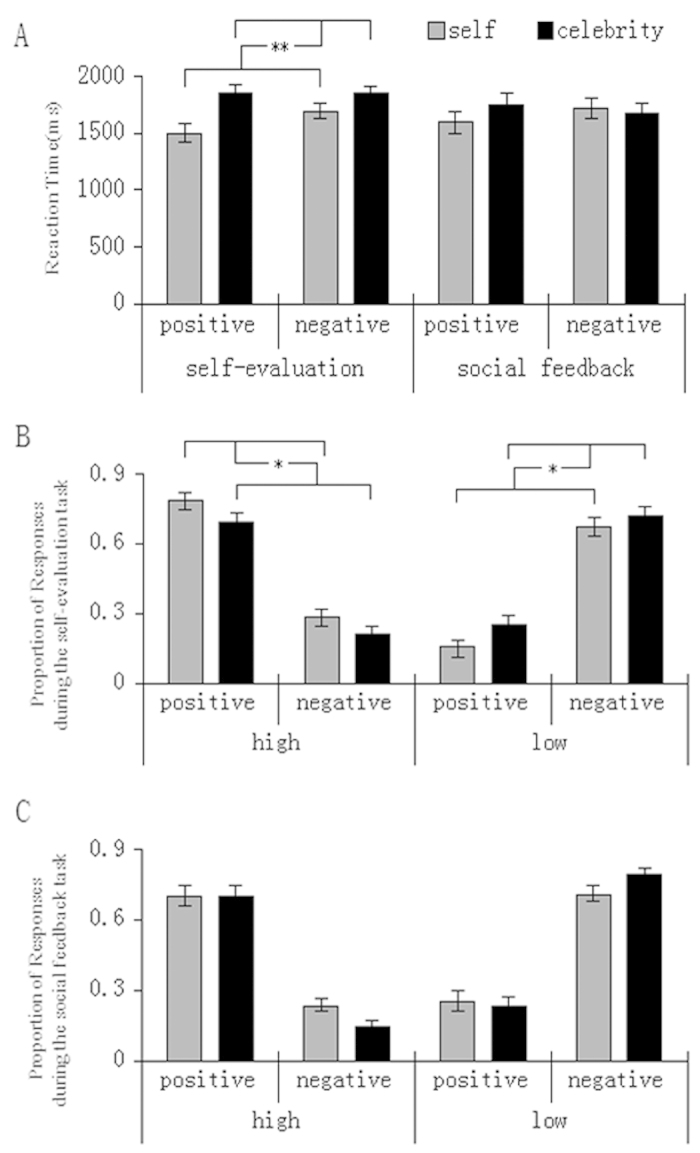
Participants’ reaction times (**A**), proportion of responses during the self-evaluation task (**B**) and proportion of responses during the social feedback task (**C**).

Behavioral data from four participants who had been excluded from fMRI data analyses due to head motion were also excluded from the behavioral data analyses. A 2 (Task: self–evaluation versus social feedback) × 2 (Target: self versus celebrity) × 2 (Valence: positive versus negative trait adjectives) repeated measures analysis of variance (ANOVA) showed a significant interaction of Task × Target on response speed, *F* (1, 24) = 7.55, *p* < 0.05. Post hoc analyses revealed that participants responded faster during evaluation of the self compared to the celebrity (*p* < 0.01), whereas response speeds did not differ between evaluations of the social feedback on the self and the celebrity (*p* = 0.51) (Fig. 2A).

Judgments were then collapsed into high (3 and 4 responses) and low (1 and 2 responses) self–relevance categories and considered into a 2 (Task: self–evaluation versus social feedback) × 2 (Target: self versus celebrity) × 2 (Valence: positive versus negative trait adjectives) × 2 (Response: high versus low) ANOVA. Results revealed a significant Response × Valence interaction, *F* (1, 24) = 87.44, *p* < 0.01, due to that participants were more likely to endorse positive information as high in self–relevance and negative information as low in self–relevance (*p* < 0.05). Results also revealed a trend toward four way interaction effect, *F* (1, 24) = 3.92, *p* = 0.059. Separate analyses showed a significant Target × Response interaction during the self–evaluation task, *F* (1, 24) = 13.66, *p* < 0.01, suggesting that participants made significantly more high response than low response to self (*p* < 0.05), and that they made significantly more low response than high response to celebrity (*p* < 0.05, Fig. 2B). However, such pattern failed to present during the social feedback task, *F* (1, 24) = 1.73, *p* > 0.1 (see Fig. 2C).

Moreover, consistent with the psychometric literature on self–esteem, participants’ trait self–esteem measures (Mean = 28.4, SD = 3.6, normally distributed by the Shapiro–Wilk test) were correlated with the endorsement of positive traits during the evaluation of the self (*r* = 0.609, *p* = 0.001), and inversely correlated with the endorsement of negative traits during the evaluation of social feedback on the self (*r* = − 0.406, *p* = 0.044).

### Neuroimaging Results

#### Neural activity related to the self during the self–evaluation task and its association with self–esteem

Evaluation of one’s own traits compared to those of a celebrity induced increased activations in the ACC (−5/35/7, *t* = 4.21, *k: number of voxels* = 282) and superior frontal gyrus (−20/35/35, *t* = 2.38, *k* = 24). Similar analyses of evaluation of one’s own positive traits compared to those of a celebrity did not show any significant activation. Participants’ trait self–esteem correlated positively with activity in several neural regions in response to evaluation of one’s own traits compared to those of a celebrity, including the middle frontal gyrus (−41/57/8, *r* = 0.68, *k* = 31), inferior frontal gyrus (−38/23/1, *r* = 0.66, *k* = 58), precuneus (−15/−50/29, *r* = 0.68, *k* = 21), cuneus (−9/−88/37, *r* = 0.64, *k* = 21), parahippocampal cortex (−26/−45/3, *r* = 0.67, *k* = 23), middle temporal gyrus (−64/−33/−10, *r* = 0.65, *k* = 34), superior temporal gyrus (−58/−51/20, *r* = 0.68, *k* = 144) and middle occipital gyrus (−27/−93/22, *r* = 0.67, *k* = 39). Meanwhile, higher levels of trait self–esteem were also associated with greater activity in the middle frontal gyrus (−44/31/36, *r* = 0.69, *k* = 68), inferior temporal gyrus (−60/−11/−19, *r* = 0.65, *k* = 29) and middle temporal gyrus (−53/−29/−9, *r* = 0.63, *k* = 29) in response to evaluations of one’s own positive traits compared to those of a celebrity (Table 1).

**Figure 3:**
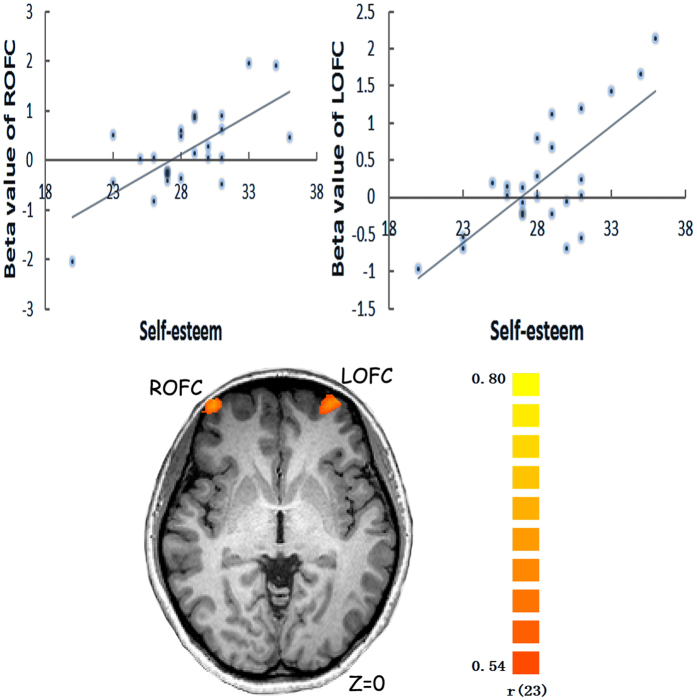
Prediction of self-esteem by attitude-related neural activity showed significant activations in the bilateral OFC in responses to evaluation of positive traits of the self compared to the celebrity (Z = 0).

Further, a whole–brain regression analysis of the neural activity in response to rating one’s own traits compared to those of the celebrity did not show any significant activation. A whole–brain regression analyses of attitude–related neural activity with self–esteem rating score as a regressor revealed significant activations in the bilateral OFC in responses to evaluation of positive traits of the self versus the celebrity (Left: −28/60/−2, *r* = 0.73, *k* = 58; Right: 40/61/3, *r* = 0.69, *k* = 32) (Fig. 3 ).

#### Neural activity related to the self during the social feedback task and its association with self–esteem

Evaluation of social feedback on the self versus the celebrity significantly activated in the ACC (−7/36/4, *t* = 5.22, *k* = 221). Similar analyses of positive social feedback on the self versus the celebrity revealed significant activations in the ACC (−9/40/4, *t* = 2.89, *k* = 22), left middle frontal gyrus (−33/38/21, *t* = 4.41, *k* = 24), posterior cingulate cortex (PCC: −54/6/60, *t* = 4.02, *k* = 60), precuneus (Left: −15/−71/46, *t* = 4.71, *k* = 279; Right: 14/−69/49, *t* = 4.11, *k* = 88), right middle temporal gyrus (34/−79/23, *t* = 4.42, *k* = 35) and middle occipital gyrus (23/−94/9, *t* = 4.28, *k* = 24) (Table 2). However, people’s trait self–esteem did not correlate with the neural activity related to social feedback on oneself.

**Figure 4:**
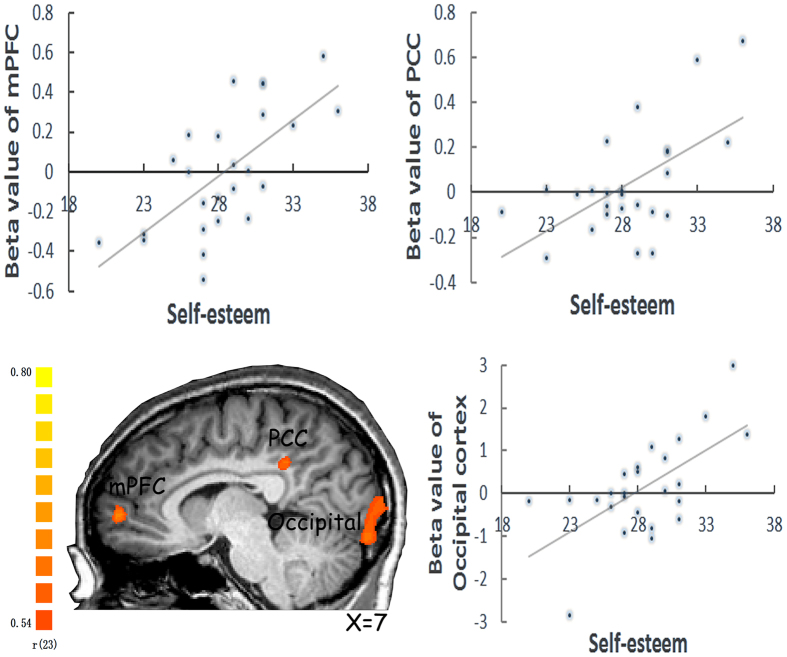
Prediction of self-esteem by attitude-related neural activity showed significant activations in the medial prefrontal cortex (mPFC), PCC and occipital cortex in responses to evaluation of positive social feedback to the self compared to the celebrity (X = 7).

Further, a whole–brain regression analysis of the neural activity in response to the evaluation of social feedback to the self versus the celebrity revealed a significant activation in the right caudate (22/−14/29, *t* = 3.49, *k* = 20). Meanwhile, a whole–brain regression analyses of attitude–related neural activity with self–esteem rating score as a regressor revealed significant activations in the ventral medial prefrontal cortex (mPFC: 9/53/3, *r* = 0.66, *k* = 25), PCC (−36/31/24, *r* = 0.63, *k* = 24) and occipital cortex (6/−92/−5, *r* = 0.63, *k* = 73) in responses to evaluation of positive social feedback to the self versus the celebrity (Fig. 4).

## Discussion

There has been less than perfect agreement within the psychological literature on the nature of self–esteem in terms of intrapersonal versus interpersonal perspectives^1^,^4^, and affective versus cognitive processes^16^,^21^,^22^,^23^. The current work examined whether and how neural activity related to self–evaluation and social feedback can be related to one’s trait self–esteem and whether and how trait self–esteem can be associated with the neural activity related to both task and attitude. Consistent with our first hypothesis, people’s trait self–esteem was positively correlated with the intrapersonal processing in OFC which has been shown to support affective processes; and consistent with our second hypothesis, their trait self–esteem was positively correlated with the interpersonal processing in mPFC/PCC which supports cognitive processes. Moreover, our fMRI results suggested that trait self–esteem predicted the task–related neural activity in the middle frontal gyrus, inferior temporal gyrus and middle temporal gyrus in response to evaluation of one’s own positive traits compared to those of a celebrity.

Interestingly, one’s self–esteem was positively associated with the affective–related neural activity in bilateral OFC, which was involved in evaluation of positive traits of the self. The orbitofrontal cortex (OFC) is an important part of the network involved in emotional processing because of its neuroanatomical connectivity with affective regions such as the amygdala, cingulate cortex, and insula^24^,^25^,^26^. Some studies have even suggested that OFC can be viewed as part of a global workspace for evaluating the affective valence of stimuli^27^,^28^. Numerous studies have shown OFC activations during affective processing, such as when receiving pleasant and painful touches^29^.OFC activation was also correlated with the amount of money received/lost in a probabilistic visual association task^30^. Damage to the OFC in humans may preclude the generation of helpful emotional information^31^, which may be associated with impairments in emotional and social behavior characterized by social inappropriateness and irresponsibility. Self–esteem is an affectively laden self–evaluation from the *intrapersonal* view^5^ and at its core, self–esteem refers to how we feel about ourselves and is inherently rooted in affective processes from the *affective* model of self–esteem^17^,^32^. Rather than being based solely on cognitive self–evaluations, self–esteem involves affective processes that may or may not be related to specific, conscious self–evaluation^5^. Therefore, the activation of OFC may be also involved in affective processing and was associated with people’s self–esteem during the self–evaluation task.

Our study also showed evidence that trait self–esteem can be also positively related to the cognitive–related neural activity in the medial prefrontal/posterior cingulate cortex during evaluation of positive social feedback about the self. Accumulating data suggests that conceiving a viewpoint of others (theory of mind), as a related form of self–projection, involves brain networks associated with the cognitive processing, including frontal lobe systems that are traditionally associated with planning, as well as medial temporal–parietal lobe systems that are associated with memory^33^. The sociometer theory proposes that self–esteem is essentially a psychological meter, or gauge, that monitors the quality of people’s relationships with others^34^. It is a person’s internal, subjective index or marker regarding the degree to which the individual is being included versus excluded by other people^4^. Thus self–esteem encompasses a cognitive processing in monitoring the relationship with others, from the *interpersonal* perspective. Moreover, trait self–esteem was also associated with activities in the occipital cortices during evaluation of positive social feedback, which was also demonstrated in a previous study showing that higher levels of state self–esteem were associated with greater activity in occipital cortex^9^.

Despite our assumption that the ‘intrapersonal’ and ‘interpersonal’ aspect of self–esteem are more affective and cognitive, respectively, we do not preclude the possibility that affective and cognitive processes are more or less present in both aspects. The only regions that associated with self–esteem were OFC and mPFC/PCC during the intrapersonal and interpersonal processes, respectively. However, other brain regions may also be involved by means of, e.g., connectivity, that was not captured in the present study. Furthermore, people’s trait self–esteem was associated with the neural activity of regions in response to positive self–reflection and social feedback. Regions that previously found to be associated with emotional self–reflection, such as anterior insula and ACC^35^,^36^, did not associate with self–esteem. One possibility is that negative self–reflection and feedback–evaluation are complicated by emotion–regulation (e.g., distraction or reappraisal) and defensive mechanisms (e.g. denial), thus less directly linked to trait self–esteem.

In addition, both ACC and PCC exhibiting high levels of activity in response to evaluation of positive social feedback about the self is remarkably similar to the default mode network (DMN), in which the total cerebral mean blood flow and oxygen uptake remain constant from a restful state to an active state^37^,^38^. Previous studies suggested that the convergence of brain regions between the DMN and that which is activated during a cognitive state raises the possibility that default modes of cognition are characterized by a shift from perceiving the external world to internal modes of cognition that simulate worlds that are separate from the one being directly experienced^33^. For instance, due to the overlap in activity between regions that are involved in self–relatedness processing and DMN regions^39^, some speak of a so–called ‘default self’ arguing that the self may be more or less identical with the resting state activity observed in DMN regions^37^,^40^,^41^. In the current study, since higher trait self–esteem was associated with significant activations in the cortical midline structures in responses to evaluation of positive social feedback about the self, we suggest that the constitution of the positive social feedback to the self in high self–esteem participants may rely on the internal resting state activity of the brain. In other words, one may then assume that processing positive social feedback in high self–esteem participants already occurs in those psychological processes associated with the brain’s resting state activity.

Eisenberg *et al.* (2011) reported greater mPFC activation in response to negative feedback words in individuals with lower state self–esteem. In the present study, higher trait self–esteem was associated with more mPFC activation in response to positive social feedback traits. Given the mPFC activation is associated with reflection upon one’s personal traits and encoding self–relevant information^42^,^43^, one may speculate that the mPFC activity may be more strongly related to the information that is being more deeply processed, independent of the valence of that information. Furthermore, the mPFC activity in response to the social feedback can predict one’s self–esteem, independent of trait or state self–esteem.

Our fMRI results also showed that task–related neural activity in memory–related brain region such as the middle frontal gyrus, inferior frontal gyrus, precuneus, cuneus, parahippocampal cortex, middle temporal gyrus, superior temporal gyrus and middle occipital gyrus in response to evaluation of one’s own traits compared to those of a celebrity could be positively related to one’s trait self–esteem. It further showed a positive correlation between one’s trait self–esteem and the neural activity in the middle frontal gyrus, inferior temporal gyrus and middle temporal gyrus related to evaluation of one’s own positive traits. Given that the self is well–developed and often used construct that promotes elaboration and organization of encoded information^44^, personality traits processed with reference to the self are better remembered than information that is processed in other ways^44^. Thus, the fMRI results in the current work are consistent with the findings of behavioral studies by showing that neural activities in the memory related brain regions such as the middle frontal gyrus, inferior frontal gyrus, precuneus, parahippocampal cortex, and middle temporal gyrus are associated with trait self–esteem.

Several limitations of this study should be noted. First, state self–esteem could reflect the feelings about the self at any moment in time^45^. Participants in the past sociometer studies rated how they felt in response to seeing each feedback word^4^,^9^. A future study could explore whether state self–esteem could be associated with both the cognitive and affective brain network during explicit self–evaluation, and to further investigate whether the cognitive network and the affective network of the brain underlying people’s perception of how they are evaluated by others would be similarly associated with state self–esteem. Second, Similar to most of the previous fMRI studies of self–evaluation, we used the celebrity–evaluation task as a control condition to rule out the impact of general evaluative processing and the impact of diversity in response selection. However, a participant might be more familiar with the self than the celebrity. This raised the issue of to what degree our results can be explained by the difference in familiarity. The previous studies have shown that coding of familiar others activated the dorsal MPFC and posterior cingulate^41^. Our results showed, however, that distinct associations between self–esteem and brain activity in responses to evaluation of the self and social feedback were evident in the OFC and medial prefrontal cortex. Although the brain activity in these brain regions was less likely to reflect the effect of familiarity, the posterior cingulate activity might be attributed to greater familiarity with the self than the celebrity. This should be clarified in the future research. Third, people learn their self–knowledge of personality through looking inward (e.g., introspection) and looking outward (e.g., feedback)^46^. Others (e.g., friends) know more than the self about aspects of personality that are observable (e.g., funny) and highly evaluative (e.g., attractive), while the self is more accurate than others for traits low in observability (e.g., neuroticism)^47^,^48^. It is likely that one’s trait self–esteem can be associated with both social feedbacks from others and self–evaluation on traits that are high in observability or low in observability. Future research can classify the personality traits into different categories to investigate this interesting topic.

In summary, our neuroimaging findings indicate associations between trait self–esteem and neural activity related to reflection on oneself and evaluation of social feedback in brain regions that are associated with affective and cognitive processes, respectively. In addition, trait self–esteem can also modulate the neural activity related to evaluation tasks on the self (but not social feedback) in a brain network that is associated with memory processes.

## Additional Information

**How to cite this article**: Yang, J. *et al.* Trait self-esteem and neural activities related to self-evaluation and social feedback. *Sci. Rep.* **6**, 20274; doi: 10.1038/srep20274 (2016).

## Figures and Tables

**Table 1:** Association between self-esteem and the neural activity related to the self during the self-evaluation task.

contrasts	Anatomical region	BA	L/R	X	Y	Z	*k*	*r*
(EPS + ENS)−(EPC + ENC)	middle frontal gyrus	10	L	−41	57	8	31	0.68
	inferior frontal gyrus	47	L	−38	23	1	58	0.66
	precuneus	31	L	−15	−50	29	21	0.68
	cuneus	19	L	−9	−88	37	21	0.64
	parahippocampal cortex		L	−26	−45	3	23	0.67
	middle temporal gyrus	21	L	−64	−33	−10	34	0.65
	superior temporal gyrus	22	L	−58	−51	20	144	0.68
	middle occipital gyrus	19	L	−27	−93	22	39	0.67
(EPS–EPC)–(ENS–ENC)	middle frontal gyrus	9	L	−44	31	36	68	0.69
	inferior temporal gyrus	20	L	−60	−11	−19	29	0.65
	middle temporal gyrus	21	L	−53	−29	−9	29	0.63

**Table 2:** Neural activity related to self during the social feedback task.

contrasts	Anatomical region	BA	L/R	X	Y	Z	*k*	*t*
(EPFS + ENFS)–(EPFC + ENFC)	ACC	24	L	−7	36	4	221	5.22
(EPFS–EPFC)–(ENFS–ENFC)	ACC	32	L	−9	40	4	22	2.89
	Middle frontal gyrus	10	L	−33	38	21	24	4.41
	PCC	30	R	1	−54	6	60	4.02
	Precuneus	7	L	−15	−71	46	279	4.71
	Precuneus	7	R	14	−69	49	88	4.11
	Middle temporal gyrus	19	R	34	−79	23	35	4.42
	Middle occipital gyrus	18	R	23	−94	9	24	4.28
